# A Fluid Dynamics-Model System for Advancing Tissue Engineering and Cancer Research Studies: Biological Assessment of the Innovative BioAxFlow Dynamic Culture Bioreactor

**DOI:** 10.3390/biomimetics10120848

**Published:** 2025-12-18

**Authors:** Giulia Gramigna, Federica Liguori, Ludovica Filippini, Maurizio Mastantuono, Michele Pistillo, Margherita Scamarcio, Alessia Mengoni, Antonella Lisi, Giuseppe Falvo D’Urso Labate, Mario Ledda

**Affiliations:** 1 Institute of Translational Pharmacology (CNR-IFT), National Research Council, Via Fosso del Cavaliere 100, 00133 Roma, Italy; giulia.gramigna@ift.cnr.it (G.G.); alessia.mengoni@ift.cnr.it (A.M.); 2 Cellex S.r.L, Piazzale delle Belle Arti 2, 00196 Roma, Italy; federica.liguori@cellex.it (F.L.); ludovica.filippini@cellex.it (L.F.); maurizio.mastantuono@cellex.it (M.M.); michele.pistillo@cellex.it (M.P.); margherita.scamarcio@cellex.it (M.S.)

**Keywords:** tissue engineering, bioreactors, osteosarcoma cell line, in vitro tumor model, 3D cell culture

## Abstract

In this study, an innovative bioreactor, named BioAxFlow, particularly suitable for tissue engineering applications, is tested. Unlike traditional bioreactors, it does not rely on mechanical components to agitate the culture medium, but on the unique fluid-dynamics behaviour induced by the geometry of the culture chamber, which ensures continuous movement of the medium, promoting the constant exposure of the cells to nutrients and growth factors. Using the human osteosarcoma cell line SAOS-2, the bioreactor’s ability to enhance cell adhesion and proliferation on polylactic acid (PLA) scaffolds, mimicking bone matrix architecture, is investigated. Cells cultured in the bioreactor showed significant improvement in cell growth and adhesion, compared to static cultures, and a more homogeneous cell distribution upon the scaffold surfaces, which is crucial for the development of functional tissue constructs. The bioreactor also preserves the osteogenic potential of SAOS-2 cells as assessed by the expression of key osteogenic markers. Additionally, it retains the tumorigenic characteristics of SAOS-2 cells, including the expression of pro-angiogenic factors and apoptosis-related genes. These results indicate that the BioAxFlow bioreactor could be an effective platform for tissue engineering and cancer research, offering a promising tool for both regenerative medicine applications and drug testing.

## 1. Introduction

The human bone is a highly dynamic tissue, which undergoes continuous structural changes: so-called remodeling. Remodeling entails continuous interplay between all the bone cells, in diverse differentiation stages, such as resident mesenchymal stem cells (MSCs)-derived osteoprogenitors, osteoblasts, osteoclasts, osteocytes, monocyte/macrophage cells and T cells [1]. An imbalance among bone cell populations is representative of both aging and pathology. Indeed, aging is defined by several biological hallmarks, including resident stem cells depletion, tissue inflammation, ECM alterations, cell senescence and metabolic dysfunction [2], while osteoporosis, a disease whose incidence increases in older people, is characterized by low bone mass and architectural deterioration, leading to enhanced bone fragility and increased fracture risk.

The operational definition of osteoporosis is based on bone mineral density (BMD) measurement [3], reflecting the porosity of the bone structure. For instance, in cortical bones, porosity is around 4% in young healthy adults and increases up to around 12% in people aged 60 [4], whereas it can get to ca. 50% in people older than 60 [5]. Nevertheless, osteoporotic fractures mostly occur in predominantly trabecular bones (i.e., proximal femurs, spine and distal radii), where fractures exert a critically important role in load transmission and energy absorption [6,7]. All the research conducted in the osteoporosis field over the last decades strongly shows a correlation between BMD and the strength of the trabecular bone, as reflected by the elastic modulus calculated from micro computed tomography (µCT) images [8].

Bone fragility associated with osteoporosis represents a socioeconomic burden on societies worldwide, as it is the root cause of most bone fractures. As an example, in France, Germany, Italy, Spain, Sweden and the United Kingdom, 2.68 million bone fractures are registered yearly. Such a figure is expected to increase 23% by 2030 (https://www.iofbonehealth.org/, accessed on 11 November 2025). Only in these countries, in 2017, fracture-related costs summed up to €37.5 billion.

These numbers suggest that novel and effective therapeutic approaches should be developed to break up the cost spiral and improve patients’ quality of life. Currently, animal models are used to study bone biology and mechanobiology or to test drugs for bone diseases. Their use, expensive, complex, and often unable to predict clinical outcomes in humans [9], is also ethically questionable and will soon be reduced to comply with the EU Directive on the 3Rs: Replacement, Reduction, and Refinement. Consequently, many alternative cell-based in vitro models have been proposed. For instance, a tissue engineering (TE) approach would be desirable for the development of sustainable and reliable bone substitutes and in vitro bone models. For this reason, we have developed a 3D platform that mimics human bone tissue in vitro, capable of recreating a microenvironment as close as possible to native tissue, through the so-called Tissue engineering-triad [10]: (1) signals, (2) biomaterials, and (3) cells. To ensure that both chemical and mechanical signals were homogenously distributed among the cultured cells, an innovative bioreactor called BioAxFlow (BAF) was used. This can gently mix the cell culture medium without the use of mechanical parts (such as impellers or rotating walls) that induce high shear stress on the cultured cells. In the past, in an effort to achieve gentle and homogeneous mass transfer within a culture chamber, microgravity-based technologies were explored. These technologies were patented for application to bioreactors (i.e., US5846817A) in the 90s, but the use of rotating mechanical components introduced several limitations to the system. In these devices, which include a vertical chamber with a series of centrally arranged rotating filters interacting with flexible membranes, the agitation and mixing process is based on fluid dynamics and therefore largely depends on the rotation speed. BAF bioreactor (patent pending), on the other hand, is able to gently mix the culture medium thanks to the fluid dynamics induced by the chamber architecture, combined with the use of a peristaltic pump. This technology, which is not dependent on microgravity or mechanical parts, makes the device a breakthrough innovation in the field of easy-to-use 3D dynamic cell culture. Historically, bone-like inorganic biomaterials (e.g., hydroxyapatite or β-tricalcium phosphate), natural polymers (e.g., ECM-based collagen type I, proteins) and synthetic polymers (e.g., polylactic-co-glycolic acid, poly caprolactone (PCL)-co-L-lactide) have been used to mimic the ECM of healthy bone [11], thus realizing the second element of the TE triad: biomimetic scaffolds. Inorganics are osteoconductive and have stiffness like that of bone but are brittle. Natural polymers suffer from high batch-to-batch variability and may elicit immune reactions. Furthermore, most polymers have insufficient mechanical strength to withstand mechanical loading. Composite materials (e.g., fiber/filler-reinforced polymeric matrices) have recently been proposed to overcome these limitations [12]. However, 3D scaffolds, prepared as millimetre-sized porous cylindrical plugs, are still difficult to prepare with a controlled and reproducible architecture from the micro to the macroscale, an important aspect that must be considered since scaffold morphology and architecture affect cell behaviour. To test the BioAxFlow bioreactor in the context of bone-tissue engineering, human osteosarcoma SAOS-2 cells were chosen as an osteoblast-like cellular model and used as the third player of the TE triad. The SAOS-2 cell line is recognized as one of the most representative osteoblast-like cell models for studying cell–material interactions in TE [13,14]. This makes them a preferred model for studying osteogenic differentiation and bone tissue dynamics. This cell line is frequently employed to investigate the effects of chemotherapeutic agents or targeted therapies on osteosarcoma, focusing on pathways regulating apoptosis (e.g., BAX/BCL2) and angiogenesis (e.g., VEGF inhibition) [15,16]. They provide a valuable platform for assessing drug resistance, sensitivity, and molecular mechanisms underlying these processes.

In this study, we propose a novel strategy for 3D cell growth by culturing SAOS-2 cells within the BioAxFlow bioreactor to evaluate their adhesion and proliferation on bone-mimicking scaffolds. SAOS-2 cells, for their robust proliferative capacity and stable phenotype, are a reliable in vitro model for assessing cell behaviour on 3D bone-mimicking scaffolds and represent a well-characterized human osteoblast-like cell line, commonly used in bone biology and TE studies. In addition, as an osteosarcoma-derived cell line, SAOS-2 cells are widely employed in oncological research to investigate tumour cell dynamics, proliferation, and response to microenvironmental cues. Their suitability for both bone tissue engineering and cancer-related studies makes them particularly appropriate for evaluating the performance of the BAF bioreactor under dynamic 3D culture conditions. These experiments aimed at assessing the compatibility and effectiveness of the bioreactor system for supporting osteoblastic cell growth and scaffold colonization, make a critical step in advancing bone tissue engineering strategies. The results demonstrate that the BioAxFlow bioreactor is a valuable platform for studying and developing functional tissue constructs in bone tissue engineering, as well as a useful tool for osteosarcoma research and cancer therapy testing.

## 2. Materials and Methods

### 2.1. Bioreactor Setup

**Figure 1:**
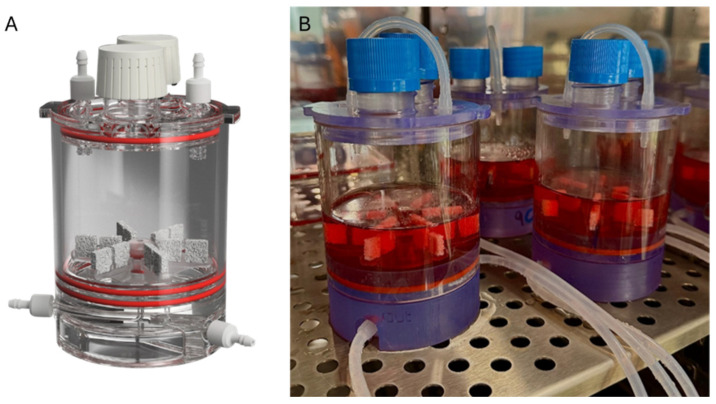
Rendering of BioAxFlow (**A**); BioAxFlow bioreactors to conduct two experiments in parallel, placed inside the CO_2_ incubator (**B**).

A commercially available bioreactor system, BioAxFlow (produced by Cellex S.r.l., Rome, Italy), was used in this study. The bioreactor comprises four main components: (1) a base (height 8 cm, Figure 1A) integrating inlet and outlet ports for controlled media flow, (2) a cylindrical chamber (diameter 8 cm, Figure 1A,B or 4 cm, not depicted), (3) scaffold stand for scaffold placement, and (4) a cap with two openings for vent caps and two sampling ports. All components were sterilized and assembled under the laminar flow hood and connected with sterile silicone tubing to a peristaltic pump (Watson Marlow 505 S, Watson-Marlow Fluid Technology Solutions, Cornwall, UK). The pump was operated using 1.6 mm ID silicone tubing, providing a stable flow rate of 80 mL/min for the 8 cm-diameter bioreactor and 40 mL/min for the 4 cm-diameter bioreactor, ensuring continuous medium recirculation through the bioreactor at the CNR-IFT laboratories (Figure 1B).

For the seeding process, a cell suspension was injected into the main chamber, where scaffolds were positioned on the appropriate scaffold stand. The peristaltic pump was then activated to initiate cell seeding, an automated process designed to maximize cell use and ensure even cell distribution across scaffolds. The scaffolds are continuously surrounded and perfused by a recirculating cell culture medium (100 mL for the 8 cm-diameter bioreactor and 30 mL for the 4 cm-diameter bioreactor) in a closed-loop system. Medium enters the bioreactor from an inlet port at the base and exits through an inner outlet, with both inlet and outlet ports featuring an internal diameter of 1.6 mm. Oxygen supply was maintained by equilibration with the incubator atmosphere (5% CO_2_, 21% O_2_), ensuring adequate Dissolved Oxygen (DO) throughout the medium. The system is compatible with placement in a standard humidified incubator at 37 °C.

### 2.2. Fluid Dynamics Simulations

The computational modeling carried out in COMSOL focuses on recapitulating the fluid dynamics characteristics of the medium flow, by investigating the velocity profiles, pressure, and Oxygen distribution within the system under consideration. In the initial stages, the model did not include the scaffolds and their holders to reduce computational complexity and establish a baseline for comparison. This allowed the analysis to concentrate on macroscale interactions, such as mixing and Oxygenation phenomena. In a subsequent phase, the scaffolds and their supporting structures were incorporated into the simulations to provide a more realistic description of the bioreactor configuration. The Oxygen transport model included a reaction term to account for cellular consumption within the scaffolds, enabling the evaluation of the Oxygen concentration in the regions where cells were cultured. Although adding both the scaffolds and their stand into the simulations increased the computational demand, this approach represented a suitable balance between accuracy and complexity for the purposes of the study.

More specifically, to model the media perfused within the BioAxFlow (BAF) bioreactor, COMSOL Multiphysics^®^ 6.3 (COMSOL AB, Stockholm, Sweden) was employed to streamline the construction of the initial simulation. The process began by defining global parameters, including the vessel’s diameter and height, as well as the internal channels and baffles in the base. Next, the fluid material was selected, with properties such as density and viscosity set to mimic water (1000 kg m^−3^ and 0.001 Pa s, respectively).

The key step involved specifying the physics of the model. This included defining the fluid domain and setting boundary conditions for both the chamber’s walls and the flow’s inlet and outlet. Additionally, initial values (such as for pressure or velocity) and free surface interactions were incorporated to accurately represent the system’s fluid dynamics.

The initial pressure and the initial velocity in all directions was set to zero. The motion of the fluid is governed by the Navier–Stokes equations, which can be seen as the application of Newton’s second law to fluid dynamics. For a compressible Newtonian fluid (Equation (1)), these equations take the form:

(1) $$\left(\rho \left(\frac{\vartheta u}{\vartheta t}+u\cdot \nabla u\right)=-\nabla p+\nabla \cdot \left(\mu \left(\nabla u+{\left(\nabla u\right)}^{Τ}\right)\right)-\frac{2}{3}\mu \left(\nabla \cdot u\right)I\right)+F$$

where *u* represents fluid velocity, *p* is fluid pressure, *ρ* is fluid density, and *μ* is the fluid dynamic viscosity. The terms in the equation correspond to inertial forces (first term), pressure forces (second term), viscous forces (third term), and external forces acting on the fluid (fourth term). The Navier–Stokes equations, developed by Navier, Saint-Venant, Poisson, and Stokes between 1827 and 1845, are always solved in conjunction with the continuity equation (Equation (2)):

(2) $$\frac{\vartheta \rho }{\vartheta t}+\nabla \cdot \left(\rho u\right)=0$$

The Navier–Stokes equations represent the conservation of momentum, while the continuity equation ensures the conservation of mass. Together, they form the foundation of fluid flow modeling. By solving these equations with specific boundary conditions—such as inlets, outlets, and walls—it is possible to predict the fluid’s velocity and pressure within a given geometry [17]. These solutions are crucial for understanding and optimizing fluid dynamics in systems like the BioAxFlow bioreactor.

**Figure 2:**
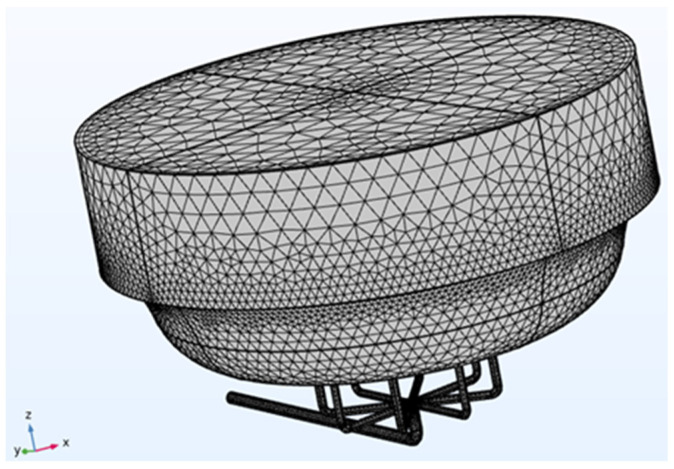
BioAxFlow (Volume = 110 mL) meshed geometry.

The next step in constructing the model involved setting up the mesh (Figure 2), or finite element network, necessary to solve the defined boundary conditions. A physics-controlled mesh with normal element size was used to minimize computational demands, such as Random Access Memory (RAM) and simulation time. The finite element method converts the problem into a system of algebraic equations, yielding approximate values for the unknowns at discrete points within the domain. This approach divides the large problem into smaller, simpler parts called finite elements. The equations for each element are then assembled into a larger system that models the entire problem [18].

The final step in the model setup involved selecting the appropriate study type, which also computed the initial conditions for time-dependent flow simulations within the vessel. Once all these steps were completed correctly, the computation could begin, with the process potentially taking several hours depending on available computational power.

### 2.3. Simulation of Oxygen Distribution

The CFD models described previously were used to simulate the spatial-temporal distribution of dissolved Oxygen. Used parameters are listed below:

D_O2_, O_2_ diffusion coefficient in aqueous media: 3 × 10^−9^ m^2^ s^−1^ [19]
Cell-normalized O_2_ consumption rate in SAOS-2 cells: 2 nmol min^−1^ 10^−6^ cells [20]
*K_m_*, Michaelis–Menten constant: 5.6 mmHg [21]
*C*_0_, O_2_ concentration at air–liquid interface: 0, 214 mol m^−3^ [19]
*K_O_*_2_, Henry’s law constant: 932.4 atm mol^−1^ L^−1^ [19]

Mass transport was estimated by advection and diffusion (Equation (3)), as described here:

(3) $$\frac{\vartheta c}{\vartheta t}+\nabla \cdot \left(-D\nabla c\right)=R-u\cdot \nabla c$$

where *c* is the dissolved Oxygen concentration, *D* is the Oxygen diffusion coefficient (m^2^ s^−1^), *R* is the reaction rate (mol m^−3^ s^−1^), and *u* is the 3D velocity field (m s^−1^) [22]. The concentration of Oxygen in the media at t = 0 was assumed to be in equilibrium with the concentration of Oxygen in the air, as described by Henry’s law (Equation (4)):

(4) $$PO_{2}=K_{O2}\cdot C_{0}$$

where *PO*_2_ is the partial pressure of Oxygen in the air and *K _O_*_2_ is the Henry’s law constant (refer to the parameters listed above, including references) and *C*_0_ represents the concentration of dissolved Oxygen in the medium at the air–liquid interface, equilibrated with the surrounding air. In the presence of cells, local Oxygen concentrations would depend on the mass transport of Oxygen in the bioreactor and the rate of Oxygen consumption by the cells. The rate of Oxygen consumption by cells was modeled using a Michaelis-Menten (MM) kinetic approach, which is widely applied to describe Oxygen uptake in mammalian cell cultures rather than cell growth [19]. The Oxygen consumption rate *R* was therefore defined as (Equation (5)):

(5) $$R=Vmax\cdot \frac{c}{c+Km}$$

where *V_max_* is the maximal Oxygen consumption rate (moles m^−3^ s^−1^), *K_m_* is the MM constant (moles m^−3^). Refer to the parameters listed above, including references. A cell-normalized Oxygen consumption rate (SAOS-2 cells) of 2 nmol min^−1^ 10^−6^ was used, along with a *K_m_* value of 5.6 mmHg. In line with previous studies on hepatocyte cultures [19], *K_m_* is treated as a constant empirical parameter describing the sensitivity of the Oxygen consumption rate to local dissolved Oxygen concentration. This simplification allows modeling Oxygen consumption without dynamically simulating changes in cell metabolism or growth. The total number of cells in BioAxFlow (d = 8 cm) was 11 million cells. *V_max_* values were calculated by multiplying the cell-normalized Oxygen consumption rate by the number of cells in the bioreactor and then dividing by the volume of the vessel (110 mL). In this study, Michaelis–Menten kinetics is applied to model Oxygen consumption by a fixed number of adherent SAOS-2 cells, independently of cell growth. Vmax values are computed based on the actual cell numbers in the bioreactor or on the scaffolds for each simulation scenario. The model does not simulate cell proliferation; its aim is to predict spatial Oxygen distribution and consumption under controlled experimental conditions.

Building on these considerations, the scaffolds were incorporated to evaluate their impact on fluid dynamics and Oxygen distribution within the vessel. To reduce computational load, the simulation domain represents 1/4 of the total bioreactor volume, leveraging the system’s geometric symmetry. Given the complexity of the actual scaffold geometry, simplified scaffolds with regular, intersecting linear struts (horizontal and vertical) were used for fluid dynamic simulations. These simplified scaffolds preserved the same porosity (about 50%) as those employed in the cell culture experiments described in this study. Oxygen consumption on scaffold surfaces was applied as a surface reaction. For simulations including scaffolds, two different cell densities per scaffold were considered: 400,000 cells, representing the number of adherent cells 24 h after seeding, and 4 million cells per scaffold, used for the calculation of *V_max_*, reflecting the cell numbers observed in the experimental cultures. The maximal Oxygen consumption rate was then computed as the product of the total number of scaffolds by the number of cells per scaffold, divided by the total surface area of all scaffolds (0.01 m^2^ per scaffold). Furthermore, as the culture medium is continuously recirculated from the outlet back to the inlet through silicone tubing, which is permeable to Oxygen, it was assumed that the Oxygen concentration at the outlet is equal to that at the inlet.

### 2.4. Scaffold Fabrication

**Figure 3:**
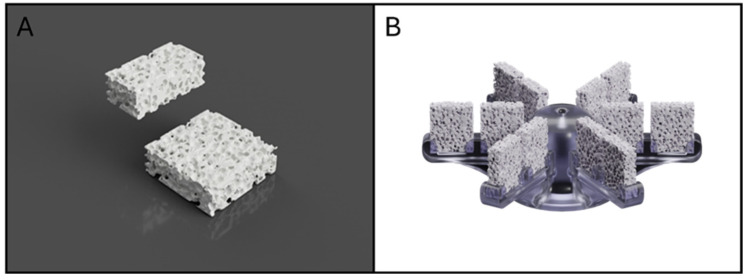
Rendering of PLA scaffold (**A**); rendering of the developed scaffold stand (**B**).

Bone inorganic matrix mimicking scaffolds have been fabricated according to the procedures described in Zenobi et al., 2023 [23] using a percentage porosity of 52.5% for reproducing the healthy bone condition. The scaffolds were designed using Meshmixer 3.5 software (v.2018, Autodesk, San Rafael, CA, USA). Briefly, the process began with a solid of 10 mm × 10 mm × 3 mm. A three-dimensional random cluster of spheres was subtracted from this solid to create a porous structure. The sphere diameter (pore size) and center-to-center distance (spacing) values were set at 700 μm [23]. The printing was performed using a commercially available fused-filament fabrication (FFF) 3D Printer (Prusa MK3S, Prusa Research, Prague, Czech Republic). The printer is equipped with a 0.4 mm brass nozzle, a direct-dive extrusion system, and a heated build plate, and operates with a layer height range of 0.05–0.30 mm and a maximum nozzle temperature of 300 °C. The printing material was a PLA filament (diameter 1.75 mm) supplied by FILOALFA (Italy). According to the manufacturer, the material has a density of 1.24 g/cm^3^, a tensile strength of 53 MPa, and a tensile modulus of 3.6 GPa. The melting point is approximately 135 °C, consistent with typical PLA grades. This PLA filament was carefully heated to a precise extrusion temperature of 205 °C, allowing it to flow through a nozzle with a diameter of 0.4 mm. During the printing process, the printer bed was consistently maintained at a temperature of 60 °C to ensure optimal adhesion and stability. Importantly, it is worth noting that all samples underwent fabrication using identical printing parameters, thereby guaranteeing the homogeneity and standardization of the produced scaffolds and control specimens. The dimensions obtained for the parallelepiped scaffolds were 10 mm × 10 mm × 3 mm (Figure 3A).

### 2.5. Scaffold Stand

During the scaffold stand fabrication procedure, a design approach aimed at optimizing the allocation of scaffolds within the bioreactor was adopted. The structure was conceived to simultaneously support twelve parallelepiped scaffolds (10 mm × 10 mm × 3 mm), ensuring efficient use of the available room. The scaffold stand, as well as the base and cap of the bioreactor, were designed using AutoCAD software (AutoCAD Fusion 360, San Rafael, CA, USA) a powerful CAD software that facilitates the creation of complex geometries and allows for precise modifications based on functional requirements (Figure 3B). Each scaffold is designed to maximize cell interaction and nutrient flow, which is essential for the success of cell culture processes. The continuous recirculation of culture medium through the bioreactor, controlled by a peristaltic pump, ensures homogeneous nutrient and Oxygen distribution around the scaffolds. The flow rate was selected based on simulations showing a homogeneous distribution of flow lines, which was also confirmed experimentally to result in uniform cell distribution on scaffolds and oxygenation. Moreover, the configuration of the scaffold stand facilitates the insertion and removal of scaffolds from the bioreactor chamber, ensuring a homogeneous distribution of environmental parameters, such as temperature and Oxygen.

### 2.6. Cell Culture

The human osteosarcoma SAOS-2 cell line (ATCC, HTB-37, Rockville, MD, USA) was cultured in high-glucose Dulbecco’s modified Eagle’s Medium (DMEM; Euroclone, Pero (MI), Italy), supplemented with 10% heat-inactivated fetal bovine serum (FBS; Euroclone, Pero (MI), Italy), 2 mM L-glutamine (Sigma, St. Louis, MO, USA), 1.0 unit·mL^−1^ penicillin (Sigma, St. Louis, MO, USA), and 1.0 mg mL^−1^ streptomycin (Sigma). Cells were maintained in plastic Petri dishes at 37 °C in a humidified incubator with 5% CO_2_. For experiments, polylactic acid (PLA) scaffolds were sterilized by immersion in 70% ethanol for 30 min, rinsed with phosphate-buffered saline (PBS), and placed within the BAF bioreactor, using an appropriately printed stand to accommodate the parallelepipedal scaffolds, as described above (for dynamic culture conditions) or placed in plastic 6-well plates for static culture controls. Importantly, the static control group was conducted using the same 3D PLA scaffold model, and not 2D monolayer cultures, to ensure a fair comparison between the two culture conditions. SAOS-2 cells were cultured at a concentration of 150,000 cells mL^−1^ or 50,000 cells mL^−1^ directly inside the bioreactor chamber of two sizes (4 cm and 8 cm in diameter, respectively), in a volume of 30 mL and 100 mL, respectively, where PLA scaffolds had been placed. Parallel static cultures were established by seeding cells onto PLA scaffolds positioned in 6-well plates at the same cell concentrations used in the dynamic condition, with a total medium volume of 6 mL per well. Cultures in both conditions were maintained for up to 10 days, and medium changes were carried out after 24 h and subsequently on days 4 and 7.

### 2.7. Cell Adhesion and Growth Analysis

Cell adhesion and growth trend was quantified by Trypan Blue exclusion assay, a colorimetric method used to assess cell viability based on membrane integrity. Viable cells exclude the dye and appear clear, whereas non-viable cells with damaged membranes take up Trypan Blue and appear blue under the microscope. SAOS-2 cells cultured within the bioreactor (dynamic conditions) or seeded onto PLA scaffolds in multi-well plates (static conditions) were harvested with 0.1% trypsin–EDTA (Sigma, St. Louis, MO, USA), washed twice with PBS and the total number of nucleated and viable cells was counted by Trypan Blue dye (0.4%) (Sigma, St. Louis, MO, USA) exclusion assay using a Bürker hemacytometer chamber. This protocol was performed at days 1, 4, 7 and 10. Each experiment was repeated three times.

### 2.8. Real-Time Quantitative RT-PCR Analysis

Total RNA was extracted from SAOS-2 cells cultured on PLA scaffolds under dynamic (bioreactor) or static conditions. Cells were harvested with 0.1% trypsin–EDTA (Sigma, St. Louis, MO, USA), washed twice with PBS, and processed after 4, 7, and 10 days. RNA isolation was performed using TRIzol Reagent (Thermo Fisher Scientific, Waltham, MA, USA) according to the manufacturer’s protocol. First-strand cDNA synthesis was performed with 1 µg of total RNA using random primers and the iScript™ cDNA Synthesis Kit (Bio-Rad, Hercules, CA, USA). Gene expression was assessed by RT-qPCR using SsoAdvanced™ Universal SYBR^®^ Green Supermix (Bio-Rad, Hercules, CA, USA) on a Bio-Rad Real-Time PCR Detection System. Reactions were conducted in triplicate, with each 20 µL reaction containing 0.5 µL cDNA template and primers at a concentration of 250 nM. The primers used are listed in Table 1.

Amplifications followed these cycling conditions:

50 °C for 2 min (Annealing)
95 °C for 10 min (DNA polymerase activation),
40 cycles at 95 °C for 15 s, and 60 °C for 1 min.

Melting curve analysis performed using Bio-Rad Dissociation Curves software (version 3.1) confirmed product specificity. This analysis generates derivative melt curves (−dF/dT) to assess amplicon specificity, highlighting the temperature at which the PCR product denatures, producing a distinct peak for each DNA species present. A single, sharp peak indicates a specific amplicon, whereas additional or broader peaks reveal nonspecific products or primer–dimer artifacts. Negative controls, omitting RNA or reverse transcriptase during cDNA synthesis, were included to rule out contamination. Relative gene expression was normalized to Glyceraldehyde-3-Phosphate Dehydrogenase (GAPDH) as an endogenous control, and data were analyzed using the 2^−ΔΔCt^ method as described by Livak and Schmittgen [24]. The amount of target was calculated using the 2^−ΔCt^ equation.

ΔCt = (average target Ct − average GAPDH Ct)(6)

Before using the ΔΔCt method for quantification, we performed a validation experiment to demonstrate that amplification efficiency for the target genes and the reference GAPDH gene was equal. The primers were designed using the GeneRunner software (version 6.0) and purchased from Eurofins; their respective sequences are reported in Table 1.

### 2.9. Confocal Laser Scanning Microscopy

SAOS-2 cells cultured for 24 h on PLA scaffolds under dynamic (bioreactor) or static conditions were repeatedly washed with PBS and fixed in 4% paraformaldehyde for 15 min and rinsed twice with PBS. The cells were stained for nuclei localization with propidium iodide (2 μg mL^−1^; Sigma). Fluorescence analyses were performed using a LEICA TCS 4D confocal microscope (Leica Microsystems, Wetzlar, Germany). Z-stack images were collected across the full thickness of the scaffolds with optical sections acquired every 5 µm andthe image reported corresponds to a representative middle section. Laser intensity, gain, and pinhole settings were optimized to minimize background and enhance signal-to-noise ratio, ensuring accurate visualization of cell distribution across the scaffold.

### 2.10. Statistical Analysis

MedCalc software (version 23.0.9; MedCalc Software Ltd., Ostend, Belgium) was used for statistical analysis, and the significance level adopted for all analyses was *p* < 0.01. For the results of the cell adhesion analysis, data were analyzed by Student’s *t*-test. For cell growth analysis, data were analyzed by the two-way ANOVA test, in which one factor is the type of treatment (Static, BAF, Static to BAF and BAF to Static), and the other factor is time (1 day, 4 days, 7 days, and 10 days).

## 3. Results

### 3.1. BioAxFlow Fluid Dynamics Simulations

The fluid dynamics simulations were performed using a flow rate of 80 mL min^−1^, and the results highlight key aspects of fluid behavior within the bioreactor under perfusion conditions.

#### 3.1.1. Velocity Field

**Figure 4:**
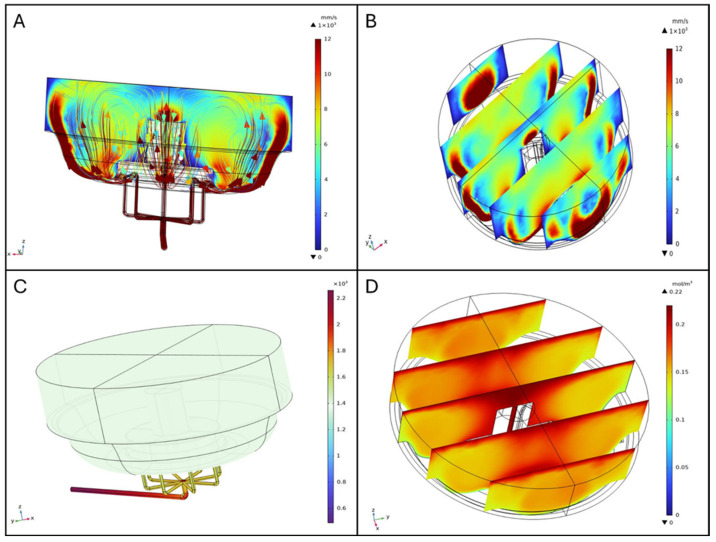
Fluid dynamics simulations (**A**) and streamline profiles, (**B**) Slice plot in ZX-plane (distributed along Y-axis), (**C**) Pressure distribution [Pa], (**D**) Oxygen distribution.

Visualizing the characterized velocity field model provides insight into how the geometry of the bioreactor influences fluid mixing dynamics. A cut-plane along the ZX-plane was used to create a cross-sectional view of the reactor, allowing for detailed observation of internal flow patterns. For clarity, all walls of the model were rendered invisible. The combined streamline plot revealed the directionality and intensity of fluid motion throughout the volume, indicating a high degree of mixing efficiency. Notably, the velocity of the fluid was higher at the inlet and outlet flow channels, as well as at the bottom of the vessel. This behavior is consistent with the bioreactor design, which promotes thorough mixing to ensure homogeneity in nutrient distribution and waste removal during perfusion (Figure 4A).

The slice plot presents a series of cross-sectional views that illustrate variations in fluid velocity across the ZX-plane and distributed along the Y-axis. Three equidistant, vertical slices spanning the diameter of the bioreactor were analyzed. The results show that fluid velocity peaks near the inlet flow channels and progressively diminishes towards the central region of the bioreactor. This gradient is attributed to the flow-dissipative effects as it spreads throughout the volume, ensuring that also those regions distant from the inlets have some level of mixing (Figure 4B).

##### Impact of Scaffolds on Flow Patterns

**Figure 5:**
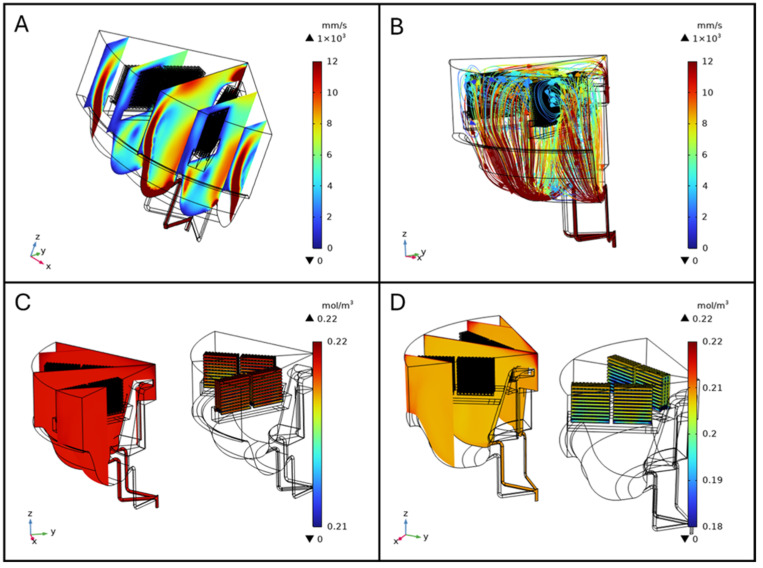
Fluid velocity slice plot in the ZX-plane distributed along Y-axis (**A**), and streamlines (**B**). Oxygen concentration distribution in the bioreactor with scaffolds seeded with 4 × 10^5^ cells per scaffold (**C**) and 4 × 10^6^ cells per scaffold (**D**).

In simulations including scaffolds, velocity plots revealed jet-like streams near the inlet and outlet channels and elevated velocities along the chamber walls, maintaining the same flow pattern as described in the one without scaffolds. The slice plot presents a series of cross-sectional views that illustrate variations in fluid velocity across the ZX-plane and distributed along the Y-axis. Five equidistant, vertical slices spanning the diameter of the bioreactor were analyzed (Figure 5A). Within the scaffold region, velocity was lower and more homogeneous, indicating a stable perfusion environment (Figure 5A). Streamline visualization showed that scaffolds redirected flow, creating localized recirculation zones, and altering streamline density. These disturbances were confined to scaffold-occupied areas, ensuring consistent exposure to the perfusion medium in those regions (Figure 5B). Wall shear stress (WSS) on scaffold surfaces was also computed from the CFD simulations; detailed maps are provided in the Supplementary Materials (Figure S1).

#### 3.1.2. Pressure Distribution

The pressure distribution analysis highlights the spatial variation in pressure within the bioreactor. The graphical representation, complemented by a color-coded legend (from blue to dark red, as pressure increases), shows that the highest pressure is located at the inlet flow channels. Beyond these regions, the pressure is uniform throughout the vessel, crucial for maintaining stable operating conditions and ensuring the integrity of the biological processes occurring within the bioreactor, as shown in Figure 4C.

#### 3.1.3. Oxygen Distribution in the Bioreactor and on Scaffolds

Oxygen distribution within the BioAxFlow bioreactor was simulated using 3D computational models. The results indicate an average Oxygen concentration of 185 μM in the vessel, with a gradient ranging from 220 μM near the air–liquid interface (dark red in Figure 4) to 130 μM near the walls (lighter red in Figure 4D). Phenomenologically, Oxygen enters the medium primarily at the air–liquid interface, where the concentration is highest, and is transported into the bulk via convection and diffusion. Cellular consumption progressively reduces Oxygen levels as the distance from the interface increases, leading to the characteristic gradient observed in Figure 4D. Regions near the inlet channels experience slightly enhanced convection, but overall, the flow remains laminar, so diffusion and consumption dominate the Oxygen distribution in the bulk. Oxygen concentration was nearly constant (~220 μM) in regions outside the scaffolds, particularly at the air–liquid interface, where no reaction occurred, consistent with inlet and air-interface boundary conditions (dark red in Figure 5C). Oxygen consumption was localized to scaffold surfaces and modeled using Michaelis–Menten kinetics to represent SAOS-2 cell metabolism, with concentrations near 220 μM in less metabolically active areas. Increasing the maximum reaction rate (V_max_), simulating higher cell densities, led to progressively lower Oxygen levels at scaffold surfaces, as expected due to the increased metabolic demand (Figure 5D). External scaffold surfaces, more exposed to flow, maintained higher Oxygen levels, close to 200 μM, but still showed a decrease with rising V_max_. Diffusive Oxygen depletion halos were observed immediately adjacent to scaffolds, while no significant consumption occurred in the bulk fluid, confirming that Oxygen consumption was confined to the scaffold–cell interfaces.

### 3.2. Analysis of Cell Adhesion to Bone-Mimicking Scaffolds Using the BioAxFlow Cell Culture System

**Figure 6:**
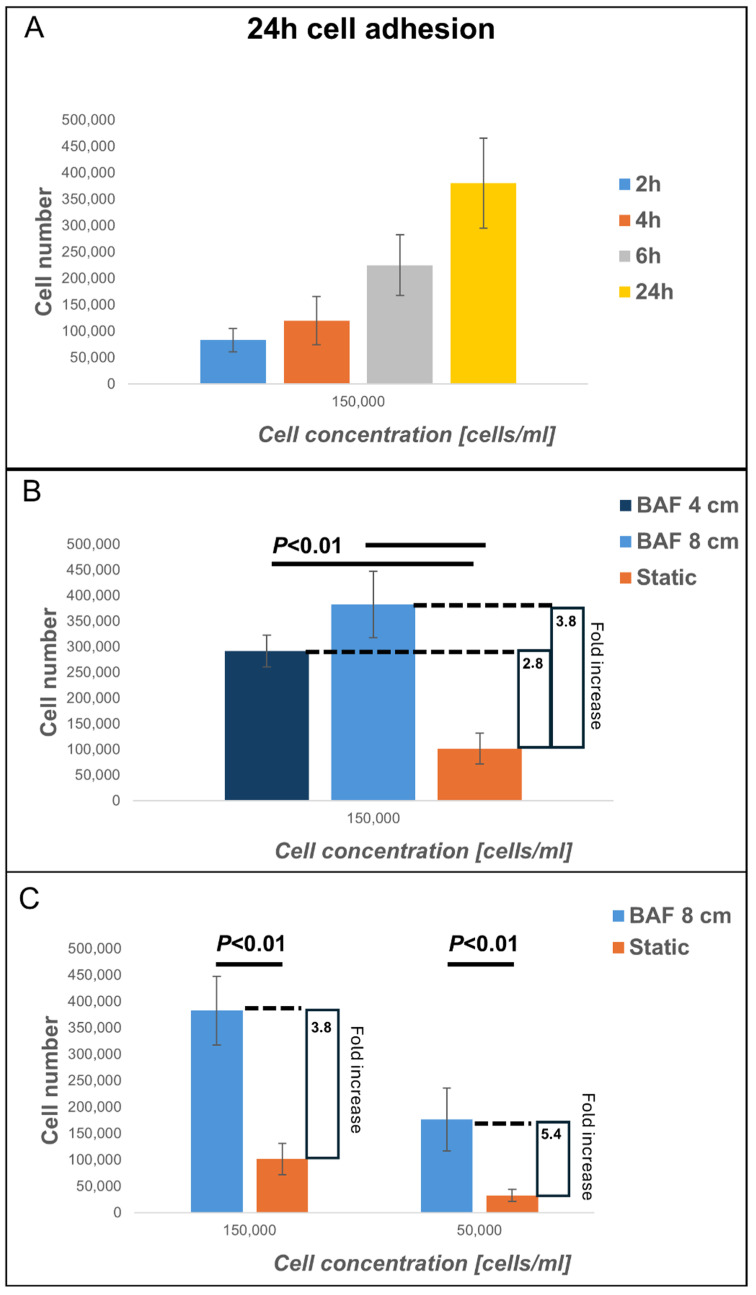
Analysis of cell adhesion at 24 h of cells cultured on PLA scaffolds under different experimental conditions: using BioAxFlow (BAF, in light blue) versus multi-well plates (static conditions, in orange). The results of the cell adhesion analysis demonstrate that the BAF bioreactor significantly enhances cell adhesion, achieving approximately a five-fold increase compared to static conditions, depending on the bioreactor’s size. (**A**) Cell adhesion over different time periods (2 h, 4 h, 6 h, and 24 h), was evaluated. (**B**) Cell adhesion comparing two different bioreactor sizes. (**C**) Cell adhesion comparing two different cell concentrations. Data are shown as mean SD. *n* = 6, for each condition (BAF 4 cm, BAF 8 cm, static conditions).

This study explored the BioAxFlow bioreactor’s potential to enhance cell colonization on biomimetic scaffolds for tissue engineering applications. Specifically, we assessed the adhesion of osteoblast-like SAOS-2 cells to PLA, bone inorganic matrix-mimicking scaffolds, after 24 h of culture in the BAF bioreactor at two initial cell concentrations: 150,000 cells mL^−1^ (Figure 6B) and 50,000 cells mL^−1^ (Figure 6C). Dynamic cultures were conducted using two BAF bioreactors in two sizes (4 cm and 8 cm in diameter, respectively), while static cultures were performed using the same 3D PLA scaffold models (and not 2D monolayers), which were positioned in standard plastic 6-well plates to ensure a consistent and comparable experimental setup between the two conditions. Cell adhesion was quantified using the Trypan Blue exclusion assay, which measured the number of nucleated and viable cells after detachment from the scaffolds used. Preliminary experiments were conducted to indirectly demonstrate the BAF bioreactor’s capability to maintain cells in suspension. The adhesion of osteoblastic SAOS-2 cells to PLA scaffolds at an initial concentration of 150,000 cells mL^−1^ over different time periods (2 h, 4 h, 6 h, and 24 h), was evaluated. The results (Figure 6A) showed that after 2h (blue bar) around 100,000 cells adhered to the analyzed scaffold, after 4h (orange bar) cell adhesion increased to around 150,000 cells, suggesting progressive attachment to the scaffold which was then confirmed at 6h post seeding (gray bar), when a significant increase in cell adhesion was observed, with over 250,000 adherent cells. Finally, at 24 h (yellow bar), cell adhesion peaked, with approximately 400,000 adherent cells, confirming the time-dependent attachment to the scaffolds as hypothesized. This continuous increase in the number of adherent cells during the first 24 h of culture indicates the ability of the BAF bioreactor to effectively maintain cells in suspension. Indeed, by ensuring that cells remain suspended in the culture medium, the bioreactor facilitates a continuous interaction with the scaffold, thereby enhancing cell colonization over time. Following these preliminary findings, we evaluated cell adhesion under dynamic and static conditions (Figure 6B). When an initial concentration of 150,000 cells mL^−1^ was seeded, the dynamic culture (using the 4 cm BAF bioreactor) resulted in 290,000 adherent cells on average, representing a statistically significant increase compared to static culture conditions (where around 101,000 adherent cells were found, on average, per scaffold). This finding indicates a 2.8-fold increase in cell adhesion onto the PLA-scaffolds when cultured within the BAF bioreactor rather than using multi-well plates. The larger 8 cm BAF bioreactor yielded even higher cell adhesion values, with an average of 380,000 cells per scaffold—a 3.8-fold statistically significant increase compared to static conditions. These results confirm that the continuous flow environment in the BAF bioreactor enhances cell colonization by improving cell suspension and exposure to nutrients and growth factors. To further analyze the impact of initial cell concentration on scaffolds’ colonization, SAOS-2 cells were also seeded at a lower concentration of 50,000 cells mL^−1^ and cultured in the 8 cm BAF bioreactor (Figure 6C). Under these conditions, dynamic culture resulted in approximately 176,000 cells adhering on analyzed scaffold, a statistically significant improvement compared to static culture, which yielded only 33,000 adhered cells per scaffold. Despite the lower initial seeding concentration, the fold increase in cell adhesion under dynamic conditions within BAF (5.4-fold) was significantly higher than that observed when using the higher 150,000 cells mL^−1^ cell concentration for seeding (3.8-fold). These findings demonstrate that the dynamic environment provided by the BAF bioreactor significantly improves cell colonization onto biomimetic scaffolds, making it a promising tool for tissue engineering applications.

### 3.3. Analysis of Cell Growth on Bone-Mimicking Scaffolds Within the BioAxFlow Cell Culture System

**Figure 7:**
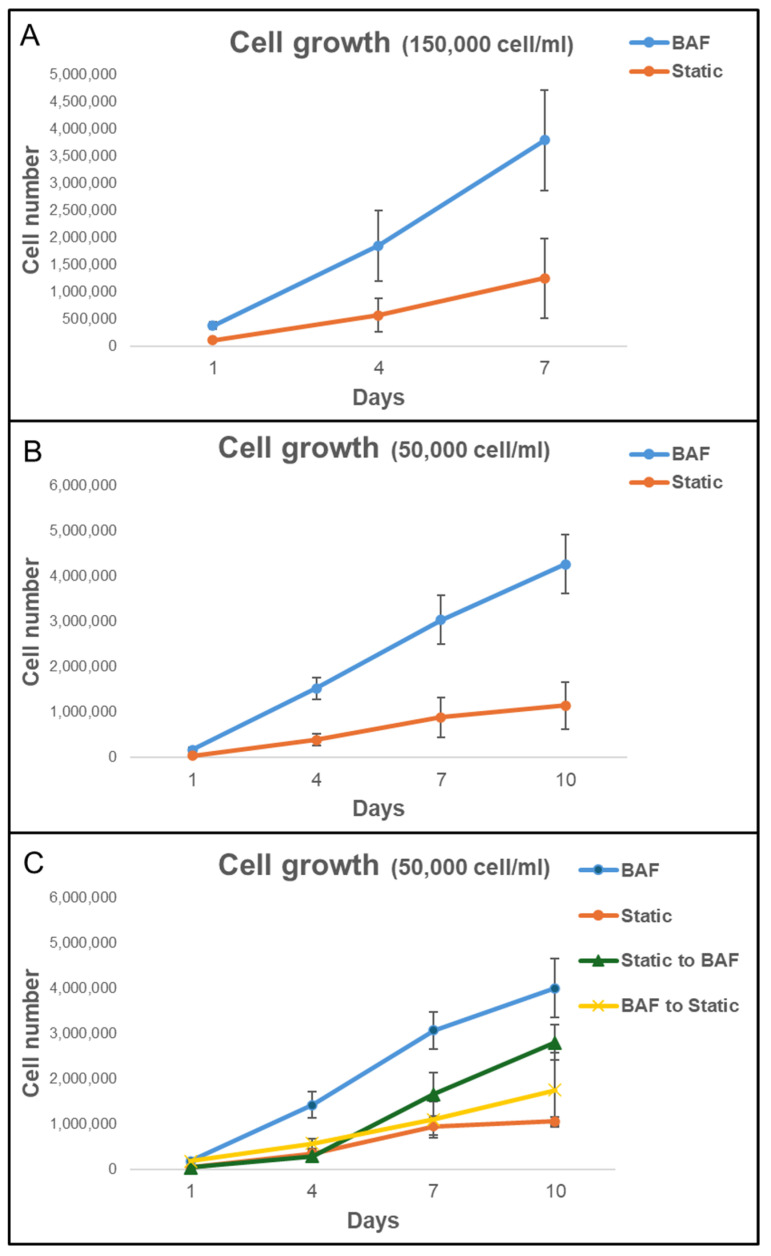
Analysis of cell growth on PLA scaffolds under different experimental conditions in the bioreactor BAF versus static conditions. The growth curves were obtained using two initial SAOS-2 cell concentrations: (**A**) 150,000 cells mL^−1^ and (**B**) 50,000 cells mL^−1^. (**C**) Analysis of cell growth on scaffold after the shift from static to dynamic conditions (Static to BAF) and from dynamic to static conditions (BAF to Static). The BAF bioreactor consistently supports a higher cell proliferation rate compared to cultivating in static conditions. Data are shown as mean SD. *n* = 6 for each experimental condition.

To further evaluate the impact of using BAF on cell growth, SAOS-2 cells were grown on bone-like scaffolds, allowing their adhesion for 24 h inside the BAF bioreactor and subsequently continued to be cultured in the same bioreactor for up to 10 days. Cell growth was quantified at days 1, 4, 7, and 10 by counting the number of viable cells on the scaffolds using the Trypan Blue exclusion assay. The growth curves were obtained using two initial SAOS-2 cell concentrations (150,000 cells mL^−1^ and 50,000 cells mL^−1^), and the experimental results obtained under dynamic (BAF bioreactor) or static conditions are shown in Figure 7. When an initial concentration of 150,000 cells mL^−1^ was used (Figure 7A), under dynamic conditions in the BAF bioreactor, the number of counted cells increased steadily from day 1, reaching approximately 4.0 million cells by day 7. By contrast, in static conditions, cell proliferation was much slower, reaching only about 1.3 million cells by day 7. By the end of the experiment, cell proliferation, when using BAF for culture, was approximately three times more than the one when using multi-well plates. This demonstrates the significant advantage derived from using dynamic culture devices in supporting rapid and efficient cell growth over a shorter timeframe. However, at the higher cell concentration of 150,000 cells mL^−1^ used for seeding, some experiments revealed the presence of non-viable cells on day 7, particularly in static conditions. This was likely due to nutrient depletion, waste accumulation, and reduced Oxygen availability in the scaffolds’ microenvironment, impairing cell viability. To avoid confounding effects associated with compromised cell vitality, the experiment at this concentration was terminated on Day 7. To investigate cell growth over an extended period, cultures were set up with a lower seeding concentration of 50,000 cells mL^−1^ (Figure 7B). Under dynamic conditions, the number of counted cells increased significantly, reaching approximately 4.2 million cells by day 10. This consistent and robust proliferation demonstrates the ability of the BAF bioreactor to sustain cell growth over a longer timeframe. In contrast, static culture conditions supported much slower growth, with the cell number reaching a maximal value of approximately 1.1 million by day 10. Thus, the BAF bioreactor supported nearly four times greater volume of cells compared to static conditions, confirming its ability to sustain high proliferation rates even with lower initial cell seeding. Statistical analysis via two-way ANOVA confirmed that, for both seeding concentrations, cell growth under dynamic conditions was significantly higher than under static conditions (*p* < 0.01). In static cultures, nutrient and Oxygen availability rely solely on passive diffusion, which becomes progressively insufficient as cell density increases, and cells penetrate deeper into the scaffold. This could induce local hypoxia, nutrient depletion, and accumulation of metabolic waste, ultimately limiting cell proliferation and contributing to the presence of non-viable cells by day 7 at the higher seeding density. In contrast, the BAF bioreactor provides a controlled dynamic environment in which continuous medium flow ensures homogeneous distribution of nutrients and Oxygen across the entire scaffold volume. Perfusion prevents the formation of nutrient- and Oxygen-depleted regions, supports efficient removal of metabolic waste, and maintains physiological conditions compatible with sustained growth.

The superior performance of the BAF bioreactor is attributed to its continuous flow system, which ensures optimal nutrient distribution, Oxygen supply, and waste removal, hence preventing reaching the plateau and decline phases of the cell growth curve observed in static culture conditions with identical experimental time length, where limited diffusion likely restricts cell viability and proliferation. These results highlight the efficacy of the BAF bioreactor in supporting enhanced cell growth and its potential as a valuable tool for 3D cell growth.

### 3.4. Effect of BioAxFlow on the Transitions from Dynamic to Static Culture on Cell Proliferation

To determine whether this effect was only due to differences in initial cell adhesion or to better culture conditions within the bioreactor, scaffolds were subjected to two experimental setups: (i) they were cultured in static conditions for 24 h (for cell seeding) and then transferred to the bioreactor for dynamic culture up to 10 days (Static to BAF); (ii) Scaffolds were cultured in the bioreactor for 24 h (for cell seeding) before being transferred to static culture conditions for up to 10 days (BAF to Static). The results, shown in Figure 7C, demonstrate that scaffolds shifted from static to dynamic conditions (Static to BAF) exhibited a statistically significant increase in cell proliferation rates compared to purely static culture. By day 10, cell counts reached approximately 2.8 million, confirming the bioreactor’s positive impact on both cell adhesion and proliferation. In contrast, scaffolds transferred from dynamic to static conditions after 24 h of seeding (BAF to Static) showed a consistent slowdown in cell proliferation rates, as expected. Before being moved to static conditions, the cell adhesion achieved with the BAF bioreactor was higher than that achieved with static culture systems. However, the growth rate slowed significantly once the scaffolds were removed from the dynamic environment and by day 10, cell counts reached approximately 1.7 million, indicating the reduced benefits of the bioreactor’s culture conditions once removed.

### 3.5. Analysis of Cell Distribution Across the Different Surfaces of the Scaffolds

**Figure 8:**
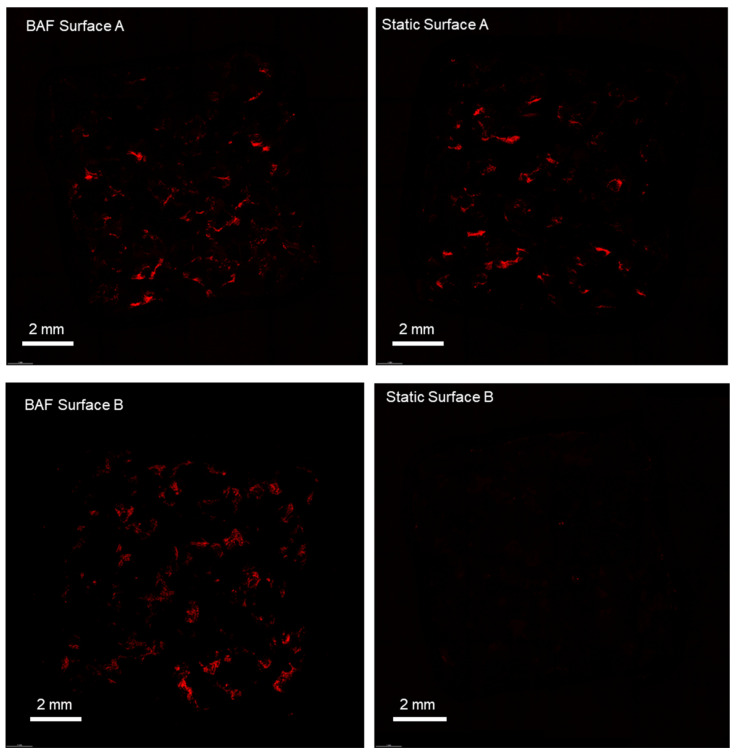
Confocal microscopy analysis of SAOS-2 cells cultured on scaffolds, under dynamic and static conditions, revealed by fluorescent dye staining (propidium iodide) of the cell nuclei. The BAF bioreactor significantly improves the colonization of PLA scaffolds, promoting uniform cell distribution across all surfaces and its fluid dynamics improves cell growth compared to static conditions. Images were taken at a magnification of 4×.

Cell colonization was further studied in terms of cell distribution across the different surfaces of the scaffolds, comparing the bioreactor-dynamic 3D culture to traditional static culture methods. To assess cell distribution, the confocal microscopy method was used; hence, the cell nuclei were stained with the fluorescent propidium iodide prior to visualization. Figure 8 reports the images of the entire surface of the analyzed scaffolds, acquired at 4× magnification. The static culture conditions do not allow for homogeneous colonization of the scaffolds, in contrast to what is observed in the dynamic culture conditions achieved using BAF. Under static culture conditions, cells primarily adhere to surface A, which is the upper surface where the cell suspension is directly deposited during seeding. In contrast, surface B, oriented towards the bottom of the plate, shows limited cell colonization. Conversely, the dynamic 3D cell culture conditions, achieved using BAF, led to the colonization of both surfaces of each scaffold. This is attributed to the fluid dynamics generated within the bioreactor, which keeps the cells in suspension, gently mixes them and permits homogeneous mass transfer.

### 3.6. Analysis of mRNA Expression in SAOS-2 Cells Grown Within the BioAxFlow Cell Culture System

#### 3.6.1. Expression of Constitutive and Osteogenic mRNA Markers

**Figure 9:**
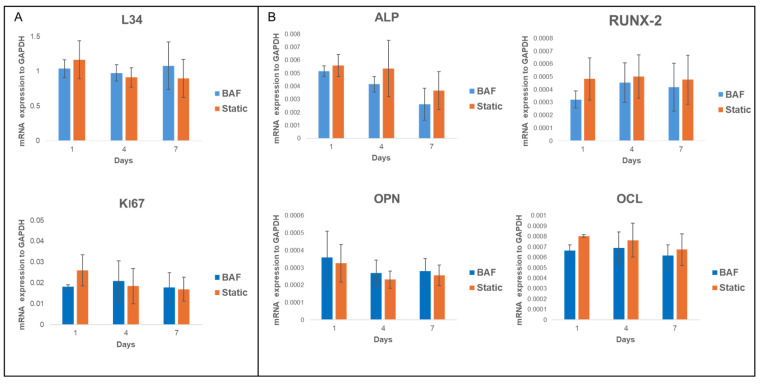
qRT-PCR analysis of the expression of (**A**) key constitutive (L34, Ki67) and (**B**) differentiation markers (RUNX2, AP, OPN, and OCL) in SAOS-2 cells cultured for up to 7 days on PLA scaffolds under dynamic BAF bioreactor conditions (BAF) versus static conditions (Static), normalized to the expression of the constitutive gene GAPDH. Data are shown as mean SD. *n* = 3 for each gene analyzed, in both experimental conditions (BAF vs. Static conditions).

To further confirm the bioreactor’s biocompatibility and evaluate its osteoconductive potential, the expression of mRNA transcripts of genes essential for biosynthetic activity and associated with osteogenic differentiation was examined. Specifically, the expression levels of key constitutive genes—Ki67 and Ribosomal Protein L34 (RPL34)—as well as osteogenic markers such as osteopontin (OPN), alkaline phosphatase (ALP), Runt-related transcription factor 2 (RUNX2), and osteocalcin (OCL) were studied by qRT-PCR assay. Ki67 is a proliferation marker involved in the cell cycle, and RPL34, a ribosomal protein, is essential during the translation process. These transcripts, highly expressed in SAOS-2 cells, are recognized as reliable indicators of correct cell functions. Their expression levels were assessed at day 1, 4 and 7 post-seeding within the bioreactor and found to be comparable to those under static conditions (Figure 9A). Noticeably, the bioreactor’s ability to maintain the osteogenic commitment of SAOS-2 cells was also validated. This commitment is a key indicator of the cells’ capacity to preserve their osteogenic potential and osteoconductive properties. The qRT-PCR analysis of osteogenic markers, conducted at day 1, 4 and 7 post-cellular seeding on PLA scaffolds within the bioreactor, showed no statistically significant differences in the expression of these markers compared to the static control (Figure 9B). These findings confirmed that the dynamic environment provided by the BAF bioreactor preserves the osteogenic phenotype of SAOS-2 cells, ensuring their ability to sustain differentiation under dynamic culture conditions.

#### 3.6.2. Expression of Tumor-Related mRNA Markers

**Figure 10:**
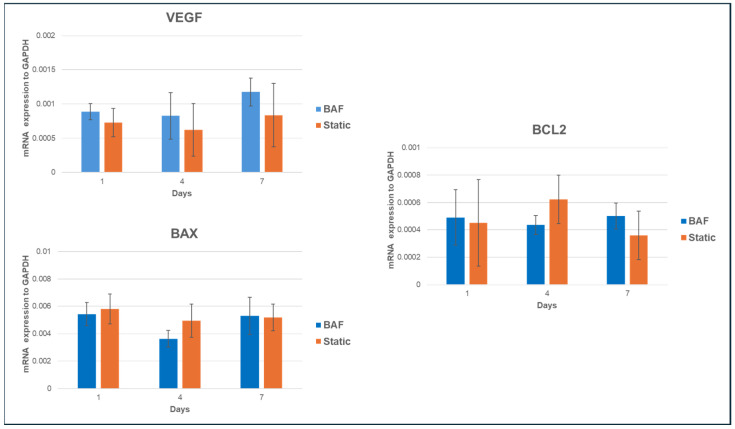
qRT-PCR analysis of VEGF, BAX, and BCL2 mRNA expression in SAOS-2 cells cultured onto PLA scaffolds under dynamic conditions compared to static ones, normalized to the expression of the constitutive gene GAPDH. Data are shown as mean SD. *n* = 3 per experimental condition, at each assessment timing (days 1, 4 and 7).

The SAOS-2 cell line is a widely used model in osteosarcoma research due to its osteoblastic characteristics and ability to mimic bone biology and cancer pathology. These cells exhibit rapid proliferation, resistance to apoptosis, express bone-specific markers, and secrete Vascular Endothelial Growth Factor (VEGF), which promotes the formation of new blood vessels, a key step in tumor growth and metastasis. To investigate the impact of dynamic culture conditions on the tumorigenic properties of SAOS-2 cells, we analyzed the mRNA expression of VEGF, Bcl-2–associated X protein (BAX), and B-cell lymphoma 2 (BCL2) genes. These genes expression was assessed by qPCR in cells cultured on PLA scaffolds under dynamic conditions (using the BAF bioreactor) and static conditions over 1, 4, and 7 days. The results, reported in Figure 10, showed no significant changes in VEGF, BAX, or BCL2 expression between dynamic and static conditions, suggesting that dynamic culture does not alter key properties related to cancer progression, such as angiogenesis or apoptosis regulation. These findings suggest that the BAF bioreactor provides a stable, biocompatible environment that maintains the cancer-like characteristics of SAOS-2 cells, making it a useful platform for in vitro studies on osteosarcoma therapies, targeting angiogenic and apoptotic pathways.

## 4. Discussion

In this study, we assessed the potential of the BioAxFlow (BAF) bioreactor to enhance the colonization, growth, and maintenance of the osteogenic and tumorigenic properties of SAOS-2 osteosarcoma cells cultured onto bone-mimicking scaffolds. These cells exhibit an osteoblast phenotype with features closely resembling those of human primary osteoblasts [25]. Compared to other osteoblast-like cell lines, SAOS-2 cells better mimic primary human osteoblast behavior when interacting with biomaterials [26]. These cells are characterized by high levels of Alkaline Phosphatase (ALP) activity, a hallmark of osteoblast differentiation, and exhibit growth factor expression and collagen production comparable to human primary osteoblasts [27]. They express bone-specific markers, such as Collagen Type I, ALP, Osteocalcin (OCL), and Osteopontin (OPN), and produce bone matrix proteins. This makes them a preferred model for studying osteogenic differentiation and bone tissue dynamics. Additionally, SAOS-2 cells are widely used in osteosarcoma and bone-related research due to their osteoblastic properties and ability to replicate key aspects of bone biology and cancer pathology. SAOS-2 cells exhibit rapid proliferation and are resistant to certain apoptotic stimuli, making them ideal for studying the growth and survival of osteo-sarcoma cells under various conditions, including the effects of potential therapeutic agents. They also exhibit features of tumor progression, such as angiogenesis and metastatic potential and secrete Vascular Endothelial Growth Factor (VEGF), which supports new blood vessel formation, a critical factor in tumor growth and metastasis [28]. Studies have highlighted the potential of targeting VEGF signaling in osteo-sarcoma, as its inhibition not only reduces angiogenesis but also promotes apoptosis, suggesting a promising therapeutic approach. This is especially relevant since VEGF overexpression is linked to worse prognosis in osteosarcoma patients [29].

Our results revealed that the BAF bioreactor led to a substantial increase in cell adhesion compared to static conditions at a seeding cell concentration of 150,000 cells mL^−1^ when using the 4 cm bioreactor. This effect was enhanced with the 8 cm bioreactor. Notably, even at a lower seeding concentration of 50,000 cells mL^−1^, dynamic conditions in the larger bioreactor (8 cm) resulted in further improvement in the average cell adhesion. This underscores the BAF system’s ability to maintain effective cell–scaffold interactions despite reduced cell availability, consistent with previous studies that have demonstrated the benefits of dynamic fluid-based culture systems for promoting efficient cell seeding and biomaterial colonization [30]. The fluid dynamics within the bioreactor, as highlighted by the COMSOL simulations, ensures uniformity in key parameters such as velocity, pressure, and gaseous distribution. Specifically, the simulations demonstrate the capability to achieve a homogeneous velocity and Oxygen distribution throughout the system, which is critical for a homogeneous distribution of cells and nutrients throughout the scaffold volume. The enhanced adhesion observed is likely due to the continuous fluid flow within the bioreactor, which improves nutrient and growth factor exchange, mimicking physiological environments more closely than static culture. This promotes greater cell interaction with biomaterial surfaces, supporting efficient scaffold seeding and better nutrient exposure. Furthermore, the improved adhesion when lower cell concentrations were used at seeding (50,000 cells mL^−1^) demonstrates the bioreactor’s efficiency and scalability. The bioreactor’s advantage extended beyond initial cell adhesion, demonstrating superior performance in supporting sustained cell proliferation over time. When a cell concentration of 150,000 cells mL^−1^ was used at seeding, dynamic culture conditions resulted in a 3-fold increase in the number of cells by day 7 as compared to static culture, which, in turn, showed reduced viability due to nutrient and Oxygen depletion, alongside waste accumulation, within the scaffold microenvironment. These findings align with previous reports emphasizing the challenges of maintaining cell vitality in static cultures, particularly at higher seeding cell densities [31,32]. Importantly, this performance advantage should be interpreted in the context of the well-known diffusion limitations intrinsic to 3D static culture, in which restricted mass transport limits nutrient supply and waste removal. By comparing the BAF system to an appropriate 3D static control, performed using the same 3D PLA scaffold models and not 2D monolayer cultures, we highlighted the specific added value of dynamic perfusion in overcoming these inherent constraints.

Importantly, at a lower seeding cell concentration of 50,000 cells mL^−1^, the BAF bioreactor enabled consistent cell growth, achieving a nearly 4-fold higher number of cells compared to static culture by day 10, highlighting its capacity to maintain optimal growth conditions over extended periods. This is consistent with findings from similar studies that show enhanced cell proliferation in bioreactors due to continuous nutrient supply, prevention of nutrient depletion, and waste accumulation that typically limit cell growth in static systems [33]. To assess whether the beneficial effects on cultured cells are merely linked to higher initial cell adhesion to the scaffolds or to better culture conditions achieved within the bioreactor, we used an approach consisting in culturing the scaffolds in static conditions for 24 h and then transferring them to the bioreactor, while other scaffolds, originally cultured in the bioreactor for 24 h, were shifted to static culture conditions. Scaffolds shifted from static to dynamic conditions (Static to BAF, in Figure 6) exhibited a significant increase in cell proliferation compared to static culture. Conversely, scaffolds moved from dynamic to static conditions (BAF to Static, in Figure 6) displayed slower growth, indicating reduced proliferation after removal from the bioreactor-dynamic environment. The recovery of cell growth rate observed when scaffolds were shifted from static to dynamic conditions underscores the bioreactor-effectiveness in enhancing proliferation even after initial suboptimal conditions. Conversely, the reduction in the growth rate observed when scaffolds were transitioned from dynamic to static culture conditions highlights the importance of sustained dynamic culture for maintaining cell proliferation and viability over time at optimal rates. By offering efficient scaffold seeding, robust cell expansion, and long-term viability, the bioreactor addresses critical challenges in scalable tissue engineering strategies. The BAF bioreactor not only supports rapid and efficient cell adhesion to the bone-mimicking scaffolds specifically designed for this study but also provides optimal conditions for sustained cell growth.

A crucial aspect of tissue engineering is the ability to achieve homogeneous cell distribution across the entire scaffold surface. When cultured using traditional static conditions, SAOS-2 cells were adhering to the surface directly exposed to the cell suspension, with minimal colonization observed on the opposite surface. In contrast, dynamic conditions facilitated uniform cell distribution across both scaffold surfaces, as demonstrated by fluorescent staining (Figure 8). This finding underscores the importance of dynamic culture systems for achieving effective cell colonization of three-dimensional scaffolds, due to the maintenance of cell suspension, which is essential for developing functional tissues for regenerative medicine.

We also investigated the effect of dynamic culture on the osteogenic potential of SAOS-2 cells. Osteogenesis involves a sequence of events across three stages: (1) proliferation, (2) extracellular matrix (ECM) deposition and maturation, and (3) mineralization of the bone ECM [34]. These phases are driven by transcription factors like RUNX2 and the activation of osteoblast-specific genes, including ALP, OCL, and OPN. ALP, an early differentiation marker, is expressed towards the end of the proliferative phase and during ECM maturation, while OPN and OCL—specific markers of the mineralization phase—are associated with advanced osteogenesis [35]. The expression of key osteogenic markers, such as RUNX2, ALP, OPN, and OCL, was assessed over time under both dynamic and static culture conditions. Our data revealed that the expression of these markers remained comparable between dynamic and static cultures, indicating that the BAF bioreactor did not compromise the osteogenic commitment of SAOS-2 cells. This result is consistent with previous studies, which suggested that bioreactors can support the osteogenic differentiation of osteoblast-like cells by maintaining physiological conditions [36].

A key aspect of our study was the evaluation of dynamic culture conditions-impact on the tumor phenotype of SAOS-2 cells. We analyzed the expression of tumor-related markers, including VEGF (a key pro-angiogenic factor), and apoptotic genes, BAX (pro-apoptotic) and BCL2 (anti-apoptotic), to assess whether dynamic culture within the BAF bioreactor would alter the tumorigenic characteristics of SAOS-2 cells. We showed that there were no significant changes in the expression of these markers under dynamic culture conditions. Specifically, VEGF expression remained stable, suggesting that dynamic conditions did not exacerbate the pro-angiogenic phenotype of the cells. Similarly, the balance between BAX and BCL2 was maintained, supporting the conclusion that the BAF bioreactor does not induce significant apoptotic stress. The lack of significant differences in VEGF, BAX, and BCL2 expression indicates that dynamic culture conditions preserve the tumor phenotype while providing a more controlled and “biomimetic” in vitro environment. This gene expression stability can be viewed positively, as it suggests that the BAF bioreactor offers a consistent and less variable platform for drug-testing applications, particularly for therapies targeting angiogenesis and apoptosis.

These findings suggest that the BAF bioreactor can be used not only in the field of tissue engineering but also as an effective platform for studying the tumorigenic characteristics of osteosarcoma cells in vitro. Its ability to maintain tumor characteristics under dynamic conditions makes it particularly valuable for drug screening and the evaluation of targeted therapeutic strategies.

Bone tissue engineering requires effective colonization of biomimetic scaffolds to establish functional constructs that integrate seamlessly with host tissue. The controlled dynamic environment of the BAF bioreactor promotes rapid cell colonization, uniform distribution across bone-mimicking scaffolds, and high cell viability. The improvements observed when using lower cell concentrations highlight its potential for efficient and cost-effective cell culture strategies, minimizing resource demands while maintaining high-quality outcomes. This advantage is particularly useful for large-scale applications and the development of complex, multilayered constructs. To optimize its performance and scalability for clinical applications, future studies should explore different flow rates and scaffold geometries. Additionally, the integration of co-culture systems and diverse cell types would provide valuable insights into the bioreactor’s versatility and broader applicability. Importantly, integrating the BAF bioreactor with advanced biomaterial technologies could significantly expand its impact. Recently developed sponge-derived bioactive glass microspheres featuring bone-like structure and controlled drug-release capabilities [37] represent an ideal class of materials that could be tested within the BAF platform. Their combination with dynamic perfusion would enable the evaluation of next-generation “smart” scaffolds capable of simultaneously supporting tissue regeneration and delivering targeted therapeutic agents. BioAxFlow can be used not only as a culture device but also as a versatile testing environment for the design and preclinical assessment of multifunctional therapeutic systems.

## 5. Conclusions

In conclusion, the BioAxFlow (BAF) bioreactor represents a powerful tool for advancing tissue engineering and cancer research. The flow generated within the culture chamber ensures continuous movement of the medium, promoting consistent cellular exposure to nutrients and growth factors, which facilitates a more efficient nutrient exchange and cellular interaction with the scaffolds, closely mimicking the conditions observed in vivo. By providing a dynamic culture environment that mimics physiological conditions, the BAF bioreactor overcomes the limitations of diffusion-based transport, such as nutrient depletion and waste accumulation, enabling rapid and sustained cell growth while maintaining cell viability and commitment. Notably, the BAF bioreactor offers a biocompatible and stable platform suitable for studying osteosarcoma models, as it preserves the tumorigenic properties of SAOS-2 cells while still enabling researchers to design specific in vitro experiments. This makes the BAF bioreactor particularly valuable for exploring cancer therapies and for both drug screening and testing therapies targeting angiogenesis and apoptosis, as required by personalized medicine. Furthermore, the system’s versatility extends beyond bone tissue engineering and osteosarcoma research, with potential applications in studying a wide range of tumor types and conducting cellular differentiation studies. Its adaptability underscores its capacity to support innovative approaches in both regenerative medicine and oncological studies. These findings pave the way for future advancements in scaffold-based tissue engineering and in vitro cancer research, facilitating the development of novel therapeutic strategies and demonstrating the potential of bioreactor technologies to revolutionize how current pre-clinical studies are conducted.

## Figures and Tables

**Table 1:** Sequence of primers used for qRT-PCR.

Target Gene	Primer Sequence	Annealing Temperature (°C)
OPN	5′-gtgtggtttatggactgagg-3′	60
	5′-acggggatggccttgtatg-3′	
Ki67	5′-tgaacaaaaggcaaagaagac-3′	60
	5′-gagctttccctattattatggt-3′	
RPL34	5′-gaaacatgtcagcagggcc-3′	60
	5′-tgactctgtgcttgtgcctt-3′	
RUNX2	5′-catcatctctgccccctct-3′	60
	5′-actcttgcctcgtccactc-3′	
ALP	5′-caatgagggcaccgtggg-3′	60
	5′-tcgtggtggtcacaatgcc-3′	
OCL	5′-cagcgaggtagtgaagag-3′	60
	5′-gaaagccgatgtggtcagc-3′	
GAPDH	5′-catcatctctgccccctct-3′	60
	5′-caaagttgtcatggatgacct-3′	
VEGF	5′-cttgggtgcattggagcct-3′	60
	5′-ctgcgctgatagacatccat-3′	
BAX	5′-tcaggatgcgtccaccaagaag-3′	60
	5′-tgtgtccacggcggcaatcatc-3′	
BCL2	5′-ggaaacttgacagaggatcat-3′	60
	5′-cggatctttatttcatgaggc-3′	

## Data Availability

The original contributions presented in this study are included in the article/Supplementary Material. Further inquiries can be directed to the corresponding authors.

## Supplementary Materials

The following supporting information can be downloaded at: https://www.mdpi.com/article/10.3390/biomimetics10120848/s1, Figure S1: Wall shear stress (WSS) distribution on scaffold surfaces in the BioAxFlow bioreactor, computed from CFD simulations.

## Abbreviations

The following abbreviations are used in this manuscript:

ALP	Alkaline Phosphatase
BAF	BioAxFlow
BMD	Bone Mineral Density
CAD	Computer-Aided Design
CFD	Computational Fluid Dynamics
MM	Michaelis–Menten
ECM	Extra Cellular Matrix
DMEM	Dulbecco’s Modified Eagle’s Medium
EDTA	Ethylen DiAmino Triacetic Acid
FBS	Fetal Bovine Serum
GAPDH	Glyceraldehyde-3-Phosphate Dehydrogenase
MSCs	Mesenchymal Stem Cells
µCT	Micro computed tomography
OPN	Osteopontin
OCL	Osteocalcin
PBS	Phosphate Buffer Saline
PCL	Poly Caprone Lactone
PLA	Poly Lactic Acid
qRT-PCR	Quantitative Reverse Transcriptase Polymerase Chain Reaction
TE	Tissue Engineering
