# Supplementary material for: A Fluid Dynamics-Model System for Advancing Tissue Engineering and Cancer Research Studies: Biological Assessment of the Innovative BioAxFlow Dynamic Culture Bioreactor
Source: Biomimetics (Basel). 2025 Dec 18;10(12):848. doi: 10.3390/biomimetics10120848 (PMC12731094; doi:10.3390/biomimetics10120848)
Supplement: Supplementary file 1 [file biomimetics-10-00848-s001.zip › biomimetics-4014154-supplementary.pdf]

biomimetics

A Fluid Dynamics-Model System for Advancing Tissue Engineering and Cancer Research Studies: Biological Assessment of the Innovative BioAxFlow Dynamic Culture Bioreactor

Giulia Gramigna [1], Federica Liguori [2], Ludovica Filippini [2], Maurizio Mastantuono [2], Michele Pistillo [2], Margherita Scamarcio [2], Alessia Mengoni [1], Antonella Lisi [1,*,†], Giuseppe Falvo D'Urso Labate [2,*,†] and Mario Ledda [1,*,†]

1 Institute of Translational Pharmacology (CNR-IFT), National Research Council, Via Fosso del Cavaliere 100, 00133 Roma, Italy; giulia.gramigna@ift.cnr.it (G.G.); alessia.mengoni@ift.cnr.it (A.M.)
2 Cellex S.r.L, Piazzale delle Belle Arti 2, 00196 Roma, Italy; federica.liguori@cellex.it (F.L.); ludovica.filippini@cellex.it (L.F.); maurizio.mastantuono@cellex.it (M.M.); michele.pistillo@cellex.it (M.P.); margherita.scamarcio@cellex.it (M.S.)
* Correspondence: antonella.lisi@ift.cnr.it (A.L.); giuseppe.falvodursolabate@cellex.it (G.F.D.L.); mario.ledda@ift.cnr.it (M.L.)
† These authors contributed equally to this work.

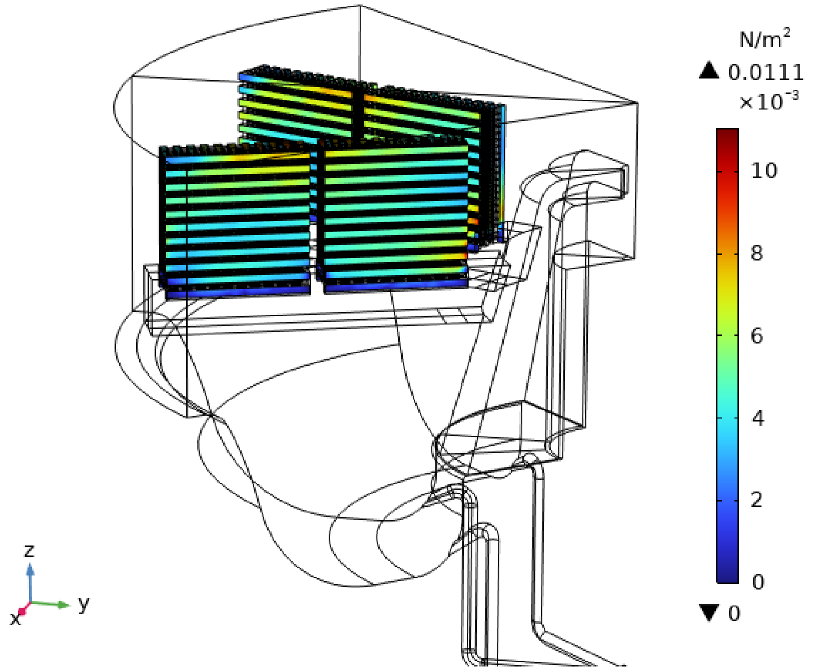

Figure S1. Wall shear stress (WSS) distribution on scaffold surfaces in the BioAxFlow bioreactor, computed from CFD simulations
